# Stump appendicitis occurred two and half years after first laparoscopic appendectomy for perforated appendicitis with abscess: A report of a case

**DOI:** 10.1016/j.ijscr.2020.01.033

**Published:** 2020-02-06

**Authors:** Noritoshi Mizuta, Takashi Nakanishi, Kozo Tsunemi

**Affiliations:** Department of Surgery, Akashi Medical Center, Akashi, Hyogo 674-0063, Japan

**Keywords:** CT, computed tomography, SIRS, systemic inflammatory response syndrome, POD, post operative day, LA, laparoscopic appendectomy, OA, open appendectomy, AA, abdominal abscess, Stump appendicitis, Laparoscopic appendectomy, Perforated appendicitis with abscess

## Abstract

**Introduction:**

The management of appendiceal abscess or phlegmon is a clinical important issue. Immediate appendectomy in these cases may be technically demanding because of the distorted anatomy and difficult to close the appendiceal stump because of the inflammation.

**Presentation of case:**

A 32-year-old female was referred to our hospital with abdominal pain. Enlarged appendix and abscess were recognized on CT scan. Preoperative diagnosis was perforated appendicitis with abscess and laparoscopic surgery was performed. The appendix was perforated and cut by stapler, but complete resection was impossible. Endoscopic transrectal drainage was performed for a pelvic abscess on the 10th POD and the patient’s condition improved. Thirty months after the surgery, however, the patient was again referred to our hospital for abdominal pain. CT scan revealed an enlarged remnant appendix. Preoperative diagnosis was stump appendicitis after the incomplete first appendectomy. Emergent second appendectomy and partial resection of the cecum were performed. The postoperative course was uneventful.

**Discussion:**

In the first operation, we mistakenly thought that the base of the appendix was cut. It was not cut, however and it remained, which was lead to stump appendicitis. Furthermore, postoperative abdominal abscess was also occurred. Immediate appendectomy for perforated appendicitis with abscess is associated with a higher morbidity. Nonsurgical treatment with drainage and/or antibiotics should be selected. Laparoscopic drainage is the useful options when CT-guided drainage is impossible.

**Conclusion:**

It is crucial to understand the correct management of perforated appendicitis with abscess to avoid serious complications.

## Highlights

• Emergent surgery for perforated appendicitis with abscess is should be carefully judged.
• It may lead to insufficient anatomical recognition or postoperative complications.
• The benefits of laparoscopic drainage for appendiceal abscess should be known.
• It is very important to understand the correct management for perforated appendicitis with abscess.

## 1 Introduction

The management of appendiceal abscess or phlegmon is controversial [1]. Immediate appendectomy in these cases may be technically demanding because of the distorted anatomy and difficult to close the appendiceal stump because of the inflammation [1]. An ileocecal resection or a right sided hemicolectomy are often needed due to the technical problems. In addition, failure to identify the base of the appendix is a major reported risk factor in the development of stump appendicitis [2]. This has been associated with significant local inflammation and other complications, such as perforation [2].

We experienced a case of stump appendicitis that required reoperation 30 months after the first laparoscopic surgery for perforated appendicitis with abscess. The purpose of the present report is to describe the importance of the correct management for perforated appendicitis with abscess. This work has been reported to in line with the SCARE criteria [3]

## 2 Presentation of a case

**Fig. 1:**
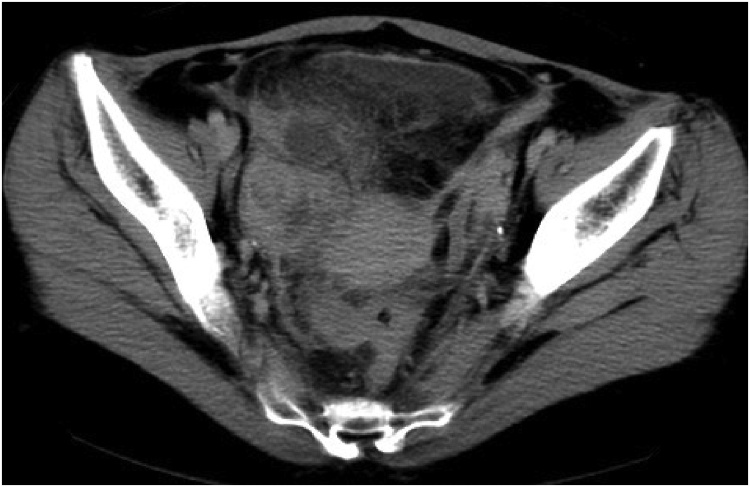
CT revealed an abscess at the right lower quadrant.

**Fig. 2:**
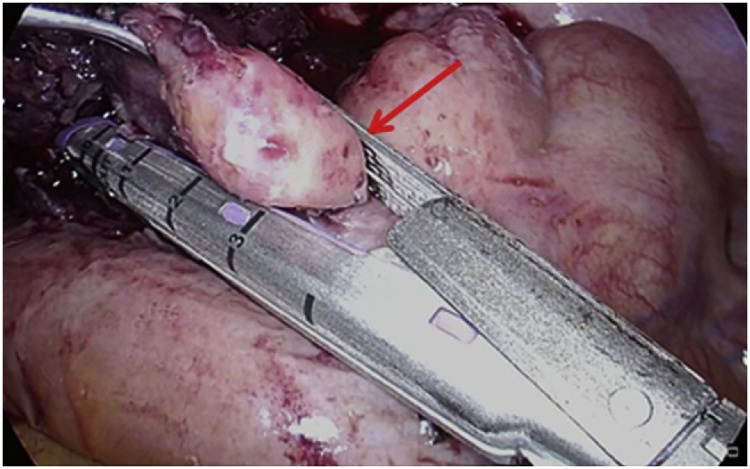
Intraoperative findings: The part considered to be the root of appendix (arrow) was cut by stapler.

A 32-year-old female had lower abdominal pain which did not improve after two weeks. Diarrhea and fever were also reported. On a visit to a clinic, no abnormal signs were recognized in the uterus or ovaries. The symptoms did not improve, so she went to another hospital, where fever and tachycardia were observed. A tumor in the right lower abdomen was detected on computed tomography (CT) scan and she was referred to our hospital for treatment. Blood pressure was 112/64, heart rate 117/min, and temperature was 40 °C. Lower abdominal pain, tenderness, rigidity and rebound tenderness were noted. White blood cell count was 23,220/μl. On CT scan, an enlarged appendix and an abscess on the pouch of Douglas were detected (Fig. 1). Preoperative diagnosis was perforated appendicitis with abscess. A peritoneal irritation sign was severe and the vital sign indicated systemic inflammatory response syndrome (SIRS). The patient was considered to getting sepsis. Therefore, emergent operation was performed laparoscopically. The cecum was covered with the omentum and the adhesion was very strong. The appendix was not seen at first, but when the omentum was peeled away, a large quantity of pus was released. As a result, the appendix was perforated and abscess cavity was formed by the omentum. The appendix was located on the medial and dorsal side. The length was about 5 cm. There was no fecalith in the abscess cavity. Because the appendix was remarkably thickened by inflammation, the part that seemed to be the base of appendix was cut by stapler (Fig. 2). Complete resection of the entire appendix was impossible, however, because of inflammation. Some parts of the appendix and abscess wall therefore remained in place. After irrigation of the abdominal cavity, a closed-type drain was inserted into the pouch of Douglas. The pathological finding was acute gangrenous appendicitis. Oral intake was restarted on the third post operative day (POD). The administration of cefmetazole was continued, but she remained febrile. Antibiotics were changed to sulbactam/ampicillin on the fourth POD. The fever had improved and the drain was serous, so the drain was removed on the seventh POD. The fever had worsened again on the eighth POD, however, and abdominal pain also became worse. CT scan was performed on the tenth POD and a large abscess was recognized in the pelvic cavity. The size was about 7.5 × 4.9 × 5.0 cm. It was near the rectum, so endoscopic transrectal drainage was performed. Ceftriaxone was continued up to the 17th POD. The fever again improved and the patient was discharged on the 18th POD.

**Fig. 3:**
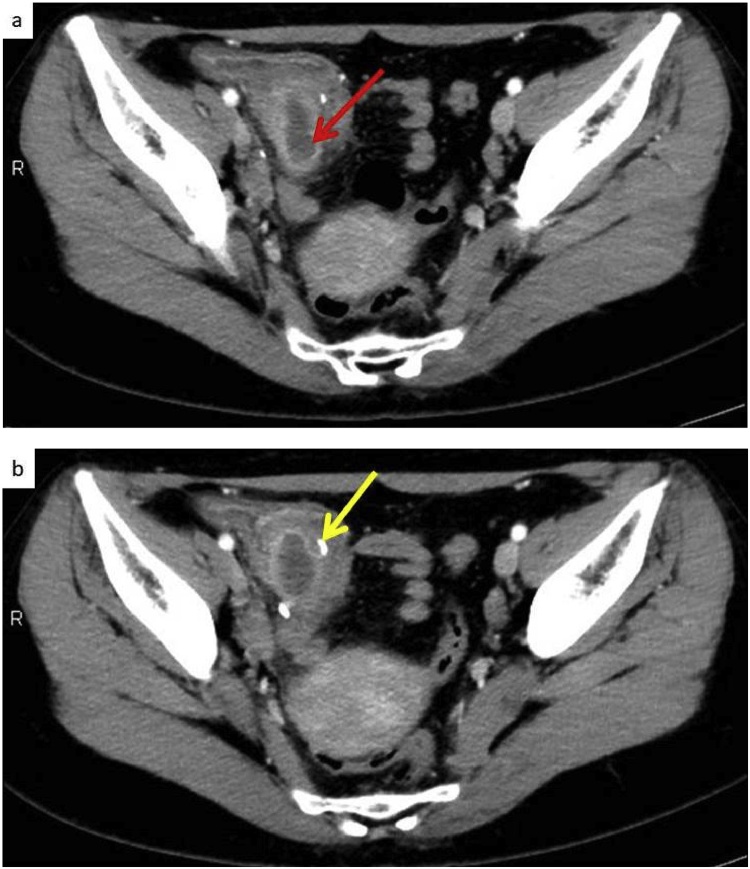
(a) CT revealed an enlarged appendix (red arrow) again. (b) The staple stump was recognized on CT (yellow arrow).

Two and a half years after the first surgery, the patient was referred to our hospital again for lower abdominal pain. CT scan showed the staple line from the appendectomy but revealed an enlarged remnant appendix (Fig. 3a,b). Preoperative diagnosis was stump appendicitis and emergent laparotomy was performed.

**Fig. 4:**
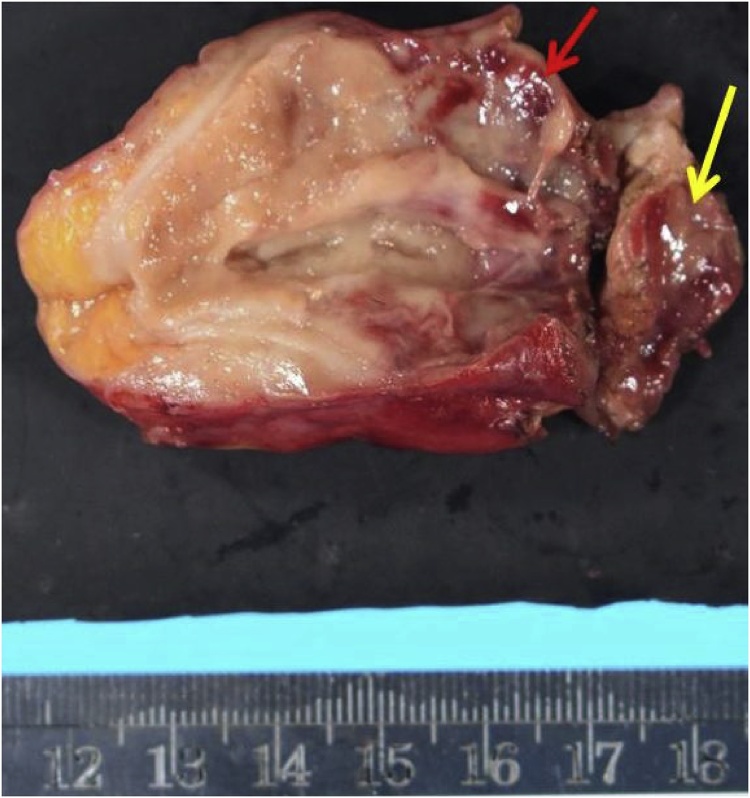
Specimen in second operation: The appendix (red arrow) and the part of cecum (yellow arrow) was resected.

After right pararectal incision, a mild adhesion of the omentum at the right lower abdomen was observed. The ascending colon and cecum were recognized and the enlarged remnant appendix was seen at the caudal side. The base of appendix was hard, so the cecum was also partially resected (Fig. 4).

The postoperative course was good and the patient was discharged on the ninth POD. The pathological finding was acute gangrenous appendicitis and there was no malignant findings.

## 3 Discussion

Laparoscopic appendectomy (LA) and open appendectomy (OA) are frequently performed for acute appendicitis. Both approaches are safe and effective and are associated with good clinical outcomes [4]. LA are associated with a lower risk of incisional infection [4,5], but higher risk of abdominal abscess (AA) than OA, especially in the case of perforated appendicitis [6]. Indication of LA for perforated appendicitis should therefore be carefully considered. In addition, in cases of appendiceal abscess or phlegmon, it is necessary to consider the indication of the appendectomy itself.

In the first operation, we mistakenly thought that the base of the appendix was cut. It was not cut, however and it remained, which was the result of uncertain anatomical recognition. Unfortunately, it lead to the stump appendicitis 30 months later. We think opening the abscess wall was also inappropriate in the first operation, which ultimately led to the postoperative abdominal abscess. The reason is why the abscess was limited in the abscess wall by the omentum, but opening the abscess wall may have further spread to the peritoneal cavity. Only drainage may have been appropriate. The postoperative course was improved by endoscopic transrectal drainage, but this disguised the severity of the appendicitis, which also seemed to improve. A second operation was ultimately necessity for treatment of the appendicitis.

Appropriate management of perforated appendicitis with abscess is clinically important issue. The patient’s abdominal pain continued for two weeks and appendiceal abscess was visible on first preoperative CT. This suggests that perforated appendicitis occurred and the abscess had already formed. Usually, CT guided or laparoscopic drainage and/or antibiotics are recommended for the patient of delayed appendicitis with abscess [7]. Immediate surgery for patients of acute appendicitis with abscess or phlegmon is technically demanding due to distorted anatomy and the difficulties in closing the appendiceal stump [1]. Furthermore, the immediate surgery often results in ileocecal resection or a right hemicolectomy and is therefore associated with a higher morbidity compared with nonsurgical treatment, such as by drainage and/or antibiotics [1]. Some reports noted the efficacy of appendectomy for complicated appendicitis (phlegmon or abcess) [8,9,10]. However, the technique needs experienced hands, therefore the standard management is nonsurgical treatment such as drainage and/or antibiotics.

Signs of severe peritonitis and SIRS were observed before the first operation, therefore we considered that the patient was getting sepsis. Therefore, we selected the emergent operation. However, it occurred postoperative complications. It was thought to be better to keep laparoscopic drainage only, even with surgical intervention. Laparoscopic drainage is one of the useful options for appendiceal abscess when CT guided drainage is impossible. The technique is performed by visualizing the imflammtory mass with laparoscope, and then entering the abscess with a laparoscopic tip, evaculating the pus, and placing a drain within the residual abscess cavity [7]. Furthermore, the technique is useful for differential diagnosis of diseases other than appendicitis, because it allows examination of the entire peritoneal cavity. On the issue of drainage only or irrigation, a prospective randomized study by Shawn et al. noted there is no advantage to irrigation of the abdominal cavity over suction alone during laparoscopic appendectomy for perforated appendicitis [11].

The interval appendectomy after successful nonsurgical treatment for appendicitis with abscess or phlegmon is controversial. Nonsurgical treatment with interval appendectomy traditionally remains as the standard of the management. However, the need for interval appendectomy has been recently challenged as the risk of recurrence, because it is rare [12]. A study by Zaza et al. noted that conservative treatment without interval appendectomy seems to be the preferred method for treatment of appendiceal mass and abscess, however they also noted that a cases such as need for percutaneous drainage presents the risk factor for the development of recurrence of appendicitis [12]. Therefore, when drainage is performed, the interval appendectomy should be considered. In adult cases, CT and colonoscopy within 4–6 weeks after the treatment is recommended to prevent pathology of right iliac fossa for example cancer or Crohn’s disease even without surgery [12].

## 4 Conclusions

We report a case of a patient with stump appendicitis after laparoscopic appendectomy for appendiceal abscess after more than two weeks of disease. The patient had immediate laparoscopic surgery, but some complications such as abdominal abscess or stump appendicitis occurred unfortunately. Nonsurgical treatment by image-guided drainage and antibiotics should be performed. The immediate appendectomy is associated with a higher morbidity, therefore it is crucial to understand the correct management of appendiceal abscess. Laparoscopic approach is one of the useful options to drainage the abscess and to observe the abdominal cavity.

## Sources of funding

Authors had no sources of funding.

## Ethical approval

IRB/Ethics Committee ruled that approval was not required for this study.

## Consent

Written informed consent was obtained from the patient for publication of this case report and accompanying images. A copy of the written consent is available for review by the Editor-in-Chief of this journal on reques.

## Author contribution

This work presented was carried out in collaboration between all authors. NM, TN, and KT defined the research theme, discussed analyses and approved the final version to be published. NM analysed the data, interpreted the results and wrote the paper.

## Registration of research studies

There is no need to register because it is a case report.

## Guarantor

Noritoshi Mizuta.

## Provenance and peer review

Not commissioned, externally peer-reviewed.

## Declaration of Competing Interest

All authors have no conflict of interest.
